# Sex-dependent effects of microglial reduction on impaired fear extinction induced by single prolonged stress

**DOI:** 10.3389/fnbeh.2022.1014767

**Published:** 2023-01-09

**Authors:** Orlando Torres-Rodriguez, Emily Ortiz-Nazario, Yesenia Rivera-Escobales, Bethzaly Velazquez, María Colón, James T. Porter

**Affiliations:** ^1^Department of Basic Sciences, Ponce Research Institute, Ponce Health Sciences University, Ponce, Puerto Rico; ^2^Department of Biomedical Sciences, Pontifical Catholic University of Puerto Rico, Ponce, Puerto Rico

**Keywords:** ventral hippocampus, fear extinction, microglia, cytokines, PTSD-like, sex-dependent

## Abstract

Single prolonged stress (SPS) is a preclinical rodent model for studying post-traumatic stress disorder (PTSD)-like behaviors. Previously we found that increased expression of the microglial marker Iba-1 in the ventral hippocampus after SPS exposure was associated with impaired fear extinction, suggesting that microglial activity contributed to the SPS-induced behavioral changes. To test this, we examined whether reducing microglia with the colony-stimulating factor 1 receptor blocker, PLX3397, in the diet would prevent the SPS-induced extinction impairment. Male rats exposed to SPS showed enhanced fear acquisition and impaired fear extinction memory. Adding PLX3397 to the diet prevented these behavioral changes. In contrast, PLX3397 did not prevent SPS from impairing fear extinction memory in the female rats. Despite the sex-dependent behavioral effects, we found a reduced number and area fraction of Iba-1+ microglia in both male and female rats suggesting that PLX3397 had similar effects on microglia in both sexes. Altogether, these results suggest that microglia contribute to the behavioral changes induced by SPS in male but not female rats.

## Introduction

Increasing clinical evidence links inflammation with posttraumatic stress disorder (PTSD) (Gill et al., 2013; Groer et al., 2015; Michopoulos et al., 2017; Wang et al., 2019; Kim et al., 2020; Katrinli et al., 2022). As the major producer of pro-inflammatory cytokines in the CNS (Kim and Joh, 2006), microglia could play a role in PTSD. Although evidence of microglial activation in PTSD is lacking, several rodent models of stress such as exposure to repeated predator stress (Wilson et al., 2013), inescapable electrical foot-shocks (Li et al., 2021), repeated social defeat (Wohleb et al., 2014), and single prolonged stress (SPS) (Lee et al., 2016; Lai et al., 2018; Cotrone et al., 2021) induce microglial activation and production of pro-inflammatory cytokines in the brain. In addition, cytokines that are produced by microglia such as interferon alpha (IFNα; Bi et al., 2016), tumor necrosis factor-alpha (TNFα; Connor et al., 1998), and interleukin 1 beta (IL-1β; Connor et al., 1998; Swiergiel and Dunn, 2007) generate abnormal fear responses or anxiety-like behaviors.

SPS is a pre-clinical model of traumatic stress that induces molecular and behavioral changes similar to those observed in PTSD (Liberzon et al., 1999) including impaired fear extinction (Knox et al., 2012; Kataoka et al., 2019), impaired cognitive flexibility (George et al., 2015, 2018; Chaby et al., 2019), and enhanced fear learning (Liu et al., 2016). In addition to the behavioral changes, exposure to SPS increases Iba-1 expressing microglia in the basolateral amygdala (BLA; Lai et al., 2018) and hippocampus (Sun et al., 2016) of male rats. Furthermore, minocycline prevented the increase in hippocampal microglia and the anxiety-like behavior and allodynia induced by SPS (Sun et al., 2016). SPS also increases microglial expression of proinflammatory P2X7 receptors and blocking P2X7 receptors prevented impaired fear extinction after SPS exposure (Torres-Rodriguez et al., 2022). Overall, the literature suggests that microglial activation contributes to the behavioral changes induced by SPS.

In this study, we further examined the role of microglia underlying PTSD-related behaviors induced by SPS exposure in male and female rats by inhibiting colony-stimulating factor 1 receptors (CSF1R). Since activation of the CSF1R signaling in microglia is required for the maintenance and development of microglia (Erblich et al., 2011; Chitu et al., 2016), the oral administration of the CSF1R inhibitor Pexidartinib (PLX3397) reduces drastically the number of microglia (Chitu et al., 2016; Merry et al., 2020). Depleting microglia with oral PLX3397 suggests that microglia support synaptic connections, neuronal plasticity, and behavioral changes (Linker et al., 2020; Liu et al., 2021; Ferrara et al., 2022). Therefore, we examined whether reducing microglia could prevent SPS-induced PTSD-related behavior. We found that oral administration of PLX3397 reduced Iba-1-expressing microglia in both male and female rats. However, PLX3397 prevented SPS from impairing fear extinction only in males suggesting a sex-dependent activation of microglia by the traumatic stress of SPS.

## Materials and Methods

### Animal subjects

All animal procedures were approved by the Institutional Animal Care and Use Committee of the Ponce Health Sciences University (PHSU) in compliance with NIH guidelines for the care and use of laboratory animals. A total of 32 adult male and 59 female Sprague-Dawley rats around postnatal day 60 were transported from the PHSU colony to a satellite facility nearby where they were individually housed on a 12/12 h light/dark schedule with free access to food and water. All behavioral experiments were carried out at the same time of the day (afternoon) to avoid the influence of the circadian rhythm. At the end of the behavioral assessments, a Wright’s stained vaginal cytological smear was taken to allow determination of the estrous cycle phase in the female rats.

### SPS

Male and female Sprague-Dawley rats approximately post-natal day 60 (P60) were pseudorandomly assigned to the SPS protocol as designed by (Liberzon and Young, 1997; Knox et al., 2012) or a non-stressed (NO-SPS) group. The SPS protocol started with 2 h of restraint stress using a disposable rodent restrainer (DecapiCone^®^; Cat. No. DC-200), followed by immediate exposure to a 20-min forced swim in a cylindrical container (20 cm × 45 cm) containing tap water at 24°C. After the forced swim, we placed the rats in a recovery cage under direct light (soft white 60 watts light bulb) as a heat source for a 10-min recovery period to allow the animals to recover from the physical stress. In the last step of our SPS protocol, we placed the rats in an anesthetic chamber (16 cm × 16 cm) and exposed them to ethyl ether (Millipore Corporation, Cat. No. EX0185-8) vapor until general anesthesia induction. Each animal received the battery of stressors contained in the SPS protocol individually. Following the SPS protocol, animals were single-housed and left undisturbed for 7 days before behavioral testing. The housing conditions for SPS and NO-SPS groups were identical.

### Diet administration of PLX3397

We pseudorandomly divided the rats into three groups (NO-SPS, SPS-ONLY, and SPS-PLX3397). All animals received the control diet (AIN-76A, Research Diets Inc., Cat. No. D15112401) for 2 days. Then, the animals belonging to the SPS-PLX3397 group received a diet switch to 290 mg/kg PLX3397 (Research Diets Inc., Cat. No. D15112401) for 3 days prior to the SPS exposure. The rats belonging to the NO-SPS, and the SPS-ONLY groups continued the control diet AIN-76A throughout the entire experiment.

### Auditory fear conditioning (AFC) and extinction (EXT)

All rats were exposed to an Auditory Fear Conditioning (AFC) and extinction (EXT) paradigm designed to test their ability to acquire and extinguish a fear memory associated with an auditory cue. During day 1, animals were exposed to a total of six tones (1 kHz, 80 dB), one unpaired tone, and five tones paired with a 0.44 mA electrical footshock with a 3-min intertrial interval (ITI) in context A. Context A consisted of a clear acrylic box with electrified grid floor (ID#46002; Ugo Basile). During day 2, animals were subjected to fear EXT which consisted of 14 tones (1 kHz, 80 dB), with a 3-min ITI in context B. On day 3, animals were presented with two tones (1 kHz, 80 dB), in context B to test their fear EXT memory. In context B, the visual, tactile, and olfactory cues were changed to avoid contextual association.

### Open field test

On day 4, we exposed the animals to a 10-min open field test (OFT) in a 94 cm × 94 cm × 44 cm arena to assess their anxiety-like behavior. No auditory cues were presented during the OFT session.

### Molecular assessments

#### Immunofluorescence staining

We collected brain tissue 24 h after behavioral testing. Brain samples were fixed, dehydrated, and embedded in paraffin. We mounted paraffin-embedded ventral hippocampal coronal slices (4 μm) onto positively charged slides. Tissue was deparaffinized in xylene and rehydrated in a descending CDA19 ethanol series. Antigen retrieval consisted of incubation with 0.01 M Citrate-EDTA solution (pH = 6.2) for 40 min followed by a 20-min incubation at room temperature. The slides were incubated overnight in a humidified chamber at 4°C with the primary antibody, Iba-1 (1:1,000; Wako Chemicals; Cat. No. 019-19741). The primary antibody was labeled with Alexa Fluor 488 Goat Anti-Rabbit (Cat. No. A-21206). A control reaction was performed without primary antibodies for each test. Tissues were covered with ProLong^TM^ Gold antifade reagent (Thermo Fischer Scientific; Cat. No. P36934) and a cover slide. Nuclei were stained with NucBlue Fixed Cell Stain (DAPI, Cat. No. 12333553). Images were taken using a Nikon Confocal Microscope A1 (Ver.4.10). Images were acquired by investigators blinded to treatment groups. Cell counting and fluorescence analyses of all images were performed using the cell-counter plug-in of the ImageJ software (NIH, USA) by investigators blinded to treatment groups. Only cells with clear DAPI-stained nuclei were counted as cells.

#### MILLIPLEX rat cytokine/chemokine magnetic panel (Cat. No. RECYTMAG-65K)

Upon sacrifice, we pseudorandomly selected either the right or the left hemisphere of the brain and stored it at −80°C until protein extraction processing. The whole hippocampus was dissected and cut into small sections using a petri dish and a scalpel. The tissue was homogenized in a low surfactant molecular grade PBS-based lysis buffer pH 7.5 containing 20 mmol/L Tris-HCl (Cat. No. 1185-53), 150 mmol/L NaCl (Cat. No. S7653), 0.05% Tween-20 (Cat. No. P9416). Protease inhibitor was diluted at 1:100 and phenylmethylsulfonyl fluoride (PMSF) was diluted at 1:1,000 in the lysis buffer. The sample was sonicated at ~3 watts for 15 s and immediately placed on ice. Then, the sample was passed through a Hamilton syringe (700 series 22 gauge) until minimal clumping. Lysates were centrifuged at 10,000 rpm for 5 min at 4°C and the supernatant was collected for protein quantification using the PIERCE BCA Protein Assay Kit (Cat. No. 23225, 23227). All hippocampal protein samples were diluted up to 1,000 μg/ml per well in the assay. Using this customized magnetic panel, we quantified the protein expression of IL-1β, TNFα, IL-6, IL-10, and IFNγ. All procedures were followed as stated by the manufacturer.

### Data analysis

All behavioral and molecular data were analyzed with Graphpad Prism (version 9.1.0, San Diego, California). Samples sizes were based on previous studies that observed differences in similar behavior or molecular analysis (Cruz et al., 2015; Criado-Marrero et al., 2017; Castillo-Ocampo et al., 2021). Animals were not excluded from the data analysis. Auditory fear was measured as the percent of time spent freezing during each 30-s tone of training and recall with ANY-maze software (Stoelting Co., Wood Dale, IL). All freezing behavioral data are presented as the mean of two trials ± SEM. Unpaired t-tests were utilized for group comparisons (Graphpad Prism version 9.1.0, San Diego, California). Two-way analysis of variance (ANOVA) for repeated measures was employed for comparisons between treatment groups over time. Sidak’s Multiple Comparisons were used for *post hoc* comparisons when appropriate. Significance was set at *p* ≤ 0.05. Details of all statistical analyses are included in a statistical table. During the 10-min OFT session, we quantified the time spent in the center, the number of entries to the center, and the total distance traveled with ANY-maze software. Two-tailed unpaired t-tests were performed for group comparisons of the acquired data (Graphpad Prism version 9.1.0, San Diego, California).

## Results

### PLX3397 prevents the SPS-induced enhanced fear acquisition in male, but not in female rats

**Figure 1:**
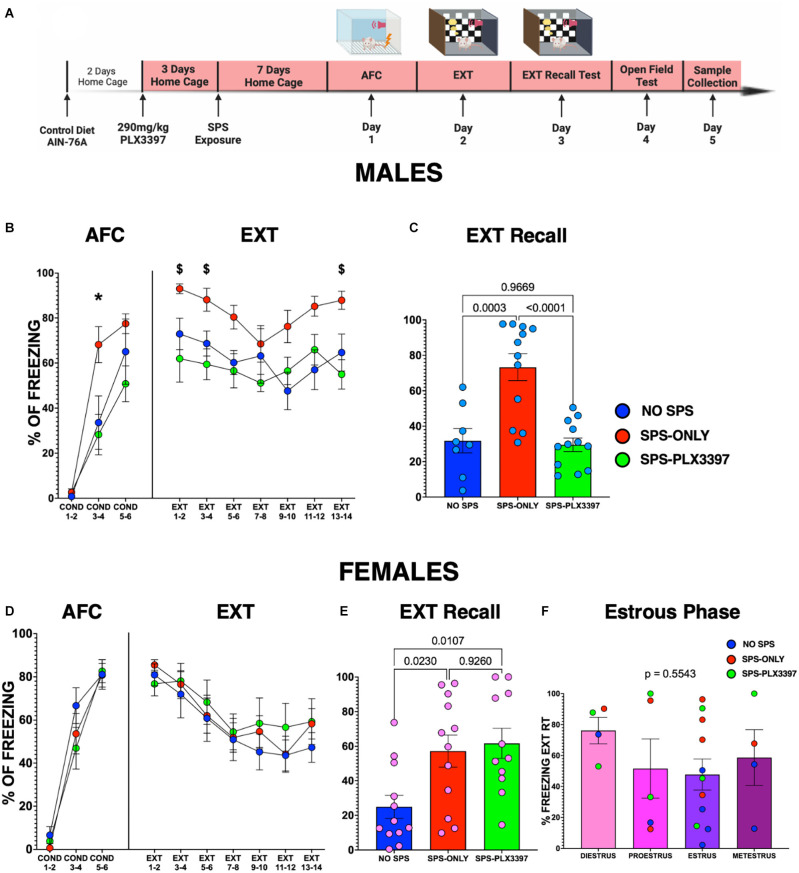
PLX3397 prevents SPS-induced impaired fear extinction in male, but not in female rats. **(A)** Experimental timeline. **(B)** Percent freezing of male rats during auditory fear conditioning (AFC), and extinction (EXT). *Denotes SPS-only is different from the other groups, *p* < 0.05. ^$^Denotes SPS-only is different from SPS-PLX3397 group, *p* < 0.05. **(C)** Percent freezing of male rats during the extinction recall test (*n* = 8 NO SPS, *n* = 12 SPS-ONLY, *n* = 12 SPS-PLX3397 males). **(D)** Percent freezing of female rats during auditory fear conditioning, and extinction training. **(E)** Percent freezing of female rats during the extinction recall test. **(F)** Percent freezing during the extinction recall test of female rats grouped by their estrous cycle phase at sacrifice (*n* = 12 NO SPS, *n* = 12 SPS-ONLY, *n* = 11 SPS-PLX3397 female rats; One-way ANOVA, Tukey’s multiple comparisons test).

First, we examined the effects of microglial reduction on auditory fear learning in male and female rats (Figure 1A). We found that the male groups did not acquire the same auditory fear on day 1 (Figure 1B; *F*_(2,87)_ = 8.120, *p* = 0.0006). The SPS-only male rats froze more than the NO-SPS male rats (*p* = 0.0195) indicating that exposure to SPS enhanced auditory fear learning. The SPS-PLX3397 male rats froze less than the SPS-only male group (*p* = 0.0009) suggesting that treatment with PLX3397 prevented the SPS enhanced fear learning in male rats. In addition, the NO-SPS and SPS-PLX3397 groups acquired similar auditory fear (*p* = 0.9999) suggesting that PLX3397 prevented the effects of the SPS without impairing normal fear learning. In contrast to the male groups, no differences in freezing were found among the female groups across the AFC session (Figure 1D; *F*_(2,96)_ = 1.105, *p* = 0.3355), suggesting that SPS did not enhance fear acquisition in the female rats.

### PLX3397 prevents SPS from enhancing fear memory and impairing extinction learning only in male rats

On day 2, we exposed the animals to 14 tones to test their recall of the fear memory and their ability to extinguish their conditioned fear response and learn that the auditory cue is not aversive. Our results show that the males belonging to the SPS-ONLY group froze more at the beginning (EXT 1–2, EXT 3–4), and the end (EXT 13–14) of the EXT session than the NO-SPS and SPS-PLX3397 male rats (Figure 1B; Day 2). This further suggests that SPS enhances fear learning and impairs extinction learning in male rats, and it could be prevented by PLX3397 oral administration. On the other hand, we found no differences in the freezing response among the female groups across the EXT session (Figure 1D; Day 2), suggesting that neither SPS exposure nor PLX3397 changes fear acquisition or extinction learning in female rats.

### PLX3397 prevents SPS from impairing fear extinction memory only in male rats

On day 3, we presented two tones to all animals to test their fear extinction recall. The male groups showed different fear extinction recall (*F*_(2,29)_ = 16.52, *p* < 0.0001). We found that SPS-ONLY male rats froze more than NO-SPS (*p* = 0.0003) and SPS-PLX3397 (*p* < 0.0001) male rats during extinction recall (Figure 1C). This suggests that SPS impairs fear extinction recall in male rats and that oral PLX3397 prevented the impairment. In addition, the NO-SPS and SPS-PLX3397 groups showed similar extinction recall (*p* = 0.9669) suggesting that PLX3397 prevented the effects of the SPS without enhancing normal fear extinction. The female groups also showed different extinction recall (*F*_(2,32)_ = 5.899, *p* = 0.0066). Female rats in the SPS-only group displayed more freezing than the NO-SPS group (*p* = 0.0230) indicating that SPS also impaired extinction recall in the female rats (Figure 1E). However, the SPS-PLX3397 group froze as much as the SPS-only group (*p* = 0.9260) during extinction recall and the SPS-PLX3397 group froze more (*p* = 0.0107) than the NO-SPS female rats. Therefore, in contrast to the male rats, oral PLX3397 did not prevent SPS from impairing fear extinction recall in the female rats.

Since the estrous cycle can affect fear extinction in female rats (Milad et al., 2009), we compared the estrous phase of the individual rats of each group at sacrifice to their freezing during extinction recall (Figure 1F). All four estrous cycle phases had some animals from each group. There was no difference in freezing during extinction recall among the groups (*F*_(3,20)_ = 0.7155, *p* = 0.5543) suggesting that the behavioral differences were not due to differences in the estrous cycle phase of the rats. However, it is important to note that since animals were sacrificed 48 h after the extinction recall test, the distribution of estrous cycle phases may have been different on other days of the behavioral protocol.

### Open field test (OFT)

**Figure 2:**
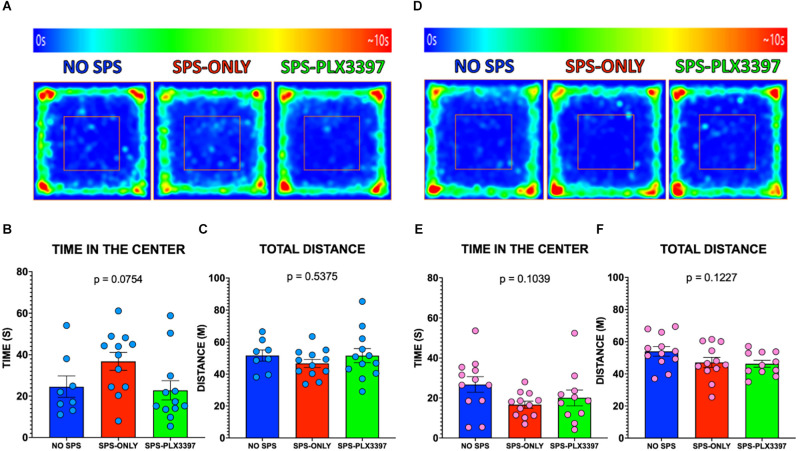
Effects of oral PLX3397 on anxiety-like behaviors in male and female rats. **(A)** Average heat maps of the center of the body of the male group during the open field testing. **(B,C)** Graphs of the time in the center and total distance traveled for the male rats. **(D)** Average heat maps of the center of the body of the female group during the open field testing. **(E,F)** Graphs of the time spent in the center and total distance traveled in the OFT for female rats. One-way ANOVA, Tukey’s multiple comparisons test.

The male groups spent similar amounts of time in the center of the OFT (*F*_(2,29)_ = 2.830, *p* = 0.0754, Figures 2A,B) and traveled similar distances during the entire OFT session (*F*_(2,29)_ = 0.6343, *p* = 0.5375, Figure 2C) suggesting that SPS and PLX3397 did not alter anxiety-like behavior. Similarly, no difference was found within the female groups in the OFT when comparing the time spent in the center (*F*_(2,32)_ = 2.432, *p* = 0.1039, Figures 2D,E). Consistent with this, no differences in the total distance traveled were found within the female groups in the OFT (*F*_(2,32)_ = 2.242, *p* = 0.1227, Figure 2F) suggesting that neither SPS nor microglial depletion exerts changes in the anxiety-like behavior of female rats.

### Oral administration of PLX3397 reduced Iba-1 positive cells in the ventral hippocampus of male and female rats

**Figure 3:**
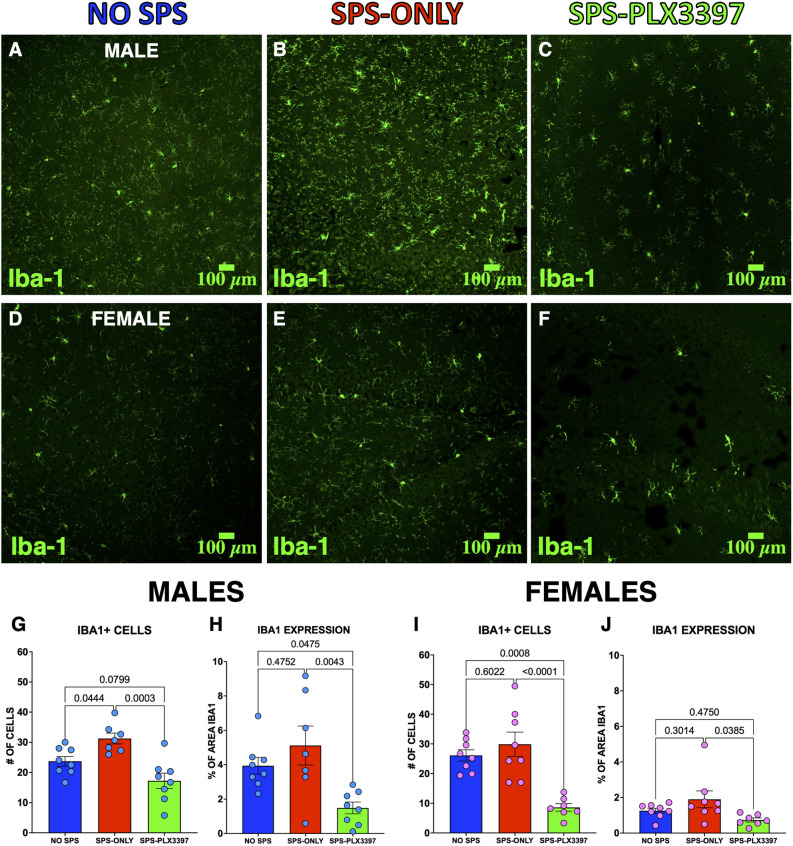
PLX3397 reduced the number of Iba-1+ cells in the ventral hippocampus in both male and female rats. **(A–C)** Representative images of Iba1-labeled ventral hippocampal slices of **(A)** NO SPS, **(B)** SPS-ONLY, and **(C)** SPS-PLX3397 male rats. **(D–F)** Representative images of Iba1-labeled ventral hippocampal slices of **(D)** NO SPS, **(E)** SPS-ONLY, and **(F)** SPS-PLX3397 female rats. **(G,H)** Iba-1+ cells and Iba1 area fraction in the ventral hippocampus of male rats. **(I,J)** Iba-1+ cells and Iba1 area fraction in the ventral hippocampus of female rats. One-way ANOVA, Tukey’s multiple comparisons test.

The inability of PLX3397 to prevent the behavioral effects of SPS could be due to the inability of 290 mg/kg PLX3397 to reduce microglia in the female rats. To test this possibility, we immunostained ventral hippocampal slices for Iba-1 to examine the effects of oral administration of PLX3397 on microglia (Figures 3A–F). We chose to examine the ventral hippocampus because SPS increases microglial expression of Iba-1 in the ventral hippocampus (Torres-Rodriguez et al., 2022) and inhibition of the ventral hippocampus impairs extinction recall (Sierra-Mercado et al., 2011; Park et al., 2020). The male groups showed differences in Iba-1 expression in both cell numbers (*F*_(2,20)_ = 11.54, *p* = 0.0005) and area fraction (*F*_(2,20)_ = 7.082, *p* = 0.0047). Ventral hippocampal slices from the SPS-PLX3397 male rats showed fewer Iba-1 positive cells (*p* = 0.0003) and reduced area fraction (*p* = 0.0043) compared to the SPS-ONLY group suggesting that PLX3397 reduced the microglia. In addition, the SPS-PLX3397 group showed a reduction in area fraction (*p* = 0.0475) and a trend towards fewer Iba-1 positive cells (*p* = 0.0799) compared to the NO-SPS male rats (Figures 3G,H). This suggests the oral administration of PLX3397 reduced microglia. In addition, more Iba-1 positive cells were found in the SPS-ONLY male group than in the NO-SPS group (*p* = 0.0444), suggesting that SPS induces changes in microglial activity in male rats.

The female groups also showed differences in Iba-1 expression in both cell numbers (*F*_(2,20)_ = 15.73, *p* < 0.0001) and area fraction (*F*_(2,20)_ = 3.569, *p* = 0.0472). In contrast to the males, the female SPS-only group did not show more Iba-1 positive cells (*p* = 0.6022) than the female NO-SPS group suggesting that SPS did not alter microglial activity in the female rats. However, PLX3397 had similar effects in the female groups since the SPS-PLX3397 female rats displayed fewer Iba-1 positive cells (*p* < 0.0001) and area fraction (*p* = 0.0385) than the SPS-only female rats (Figures 3I,J). In addition, we found fewer Iba-1 positive cells in the SPS-PLX3397 group (*p* = 0.0008) compared to the NO-SPS female rats. These results validate that the oral administration of 290 mg/kg of PLX33937 produced similar reductions in microglia in male and female rats and suggest that the development of stress-induced extinction impairment involves different mechanisms in male and female rats.

### Microglial depletion alters hippocampal pro-inflammatory cytokine expression in female rats

**Figure 4:**
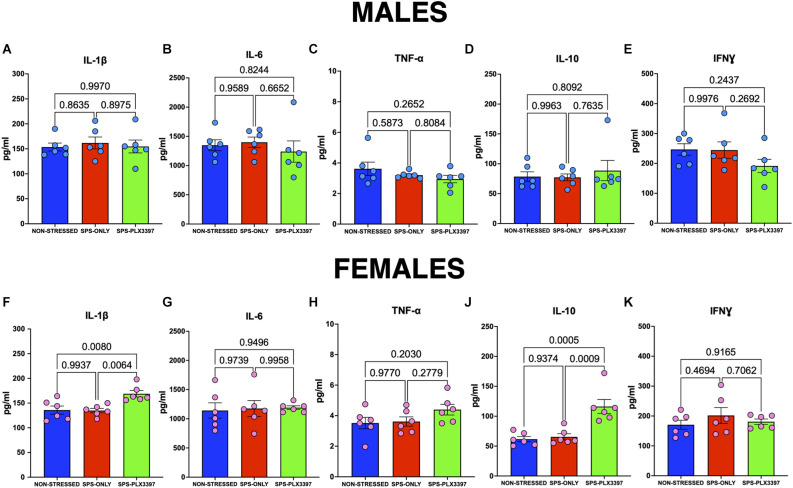
PLX3397 treatment increases hippocampal IL-1β and IL-10 in females, but not in male rats**. (A–E)** Hippocampal cytokine expression of male rats. **(F–K)** Hippocampal cytokine expression of female rats. One-way ANOVA, Tukey’s multiple comparisons test.

All animals were sacrificed the day after the OFT and hippocampal tissue was collected. To determine whether microglial depletion altered hippocampal cytokine expression, we quantified hippocampal TNFα, IL-1β, IL-6, IL-10, and INFγ pro-inflammatory cytokines using a customized MILLIPLEX Rat Cytokine/Chemokine Magnetic Panel (Cat. No. RECYTMAG-65K). Our results showed no changes in hippocampal cytokine expression in the SPS-only male groups compared to both NO-SPS or SPS-PLX3397 male groups (Figure 4) suggesting that neither SPS nor PLX3397 altered these cytokines at this time point. In contrast, we found increased hippocampal IL-1β in PLX3397-treated females when compared to the NO-SPS (*p* = 0.0080) or the SPS-only (*p* = 0.0064) female rats (Figure 4F). In addition, increased hippocampal IL-10 was found in PLX3397-treated females when compared to NO-SPS (*p* = 0.0005) or the SPS-only (*p* = 0.0009) female rats (Figure 4J).

### Oral administration of PLX3397 alone does not disrupt fear expression but produces anxiogenic behavior in female rats

**Figure 5:**
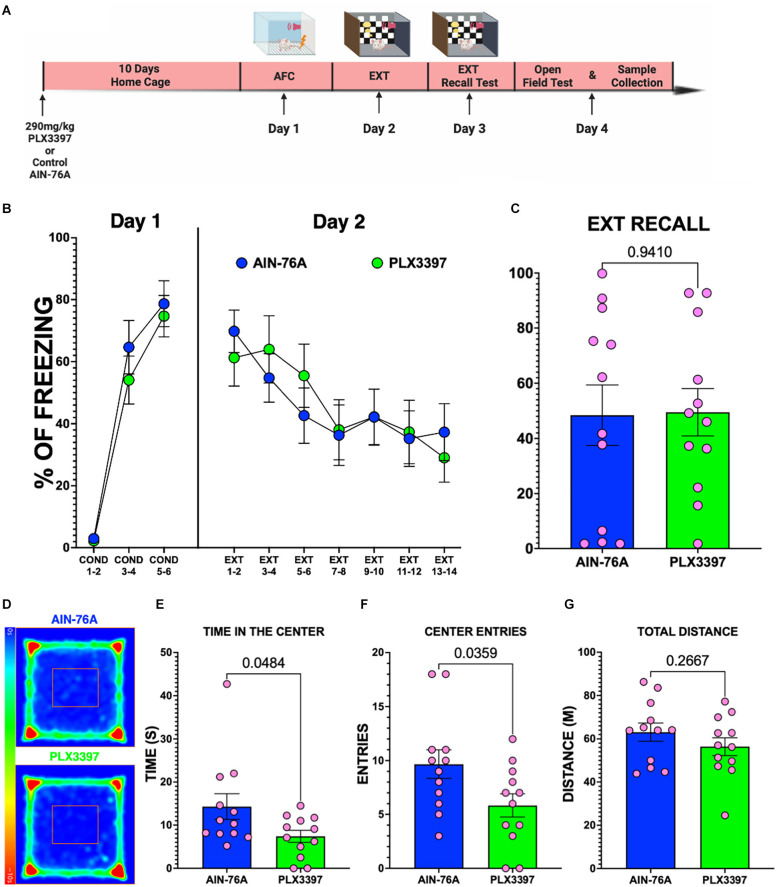
Oral administration of PLX3397 alone does not disrupt fear, but reduces the time spent in the center of the OFT in female rats. **(A)** Experimental timeline. **(B)** Percent freezing of female rats across the behavioral paradigm. **(C)** Percent freezing during EXT-Recall on Day 3 of female rats (unpaired t-test, two-tailed; *n* = 12 female rats per group). **(D)** Average heat maps of the center of the body of female groups during the OFT. **(E–G)** Graphs of the time in the center, number of entries to the center of the arena, and the total distance traveled for the female rats (unpaired t-test, two-tailed; *n* = 12 female rats per group).

Since the oral PLX3397 did not prevent SPS from impairing fear extinction recall in female rats, it is possible that PLX3397 alone impairs fear extinction memory in female rats. Therefore, we examined the effects of PLX3397 on fear conditioning and extinction in non-stressed female rats (Figure 5A). Our results showed that both PLX3397-treated and control diet-treated female rats displayed similar fear during AFC on Day 1 (Figure 5B) and during extinction on Day 2 (Figure 5B) indicating that PLX3397 did not cause significant changes in either fear acquisition or extinction. In addition, female rats fed the PLX3397 diet exhibited similar freezing during extinction recall as those fed the AIN-76A control diet (*p* = 0.9410) indicating that PLX3397 did not impair fear extinction recall in female rats (Figure 5C). On the other hand, PLX3397-treated female rats spent less time (Figures 5D,E) and entered fewer times into the center of the OFT (Figure 5F) than the female rats fed the AIN-76A control diet. PLX3397 treatment did not change the total distance traveled during the OFT session suggesting that the less time spent in the center of the OFT was not due to reduced movement (Figure 5G). This suggests that oral PLX3397 may produce anxiogenic behaviors in female rats.

## Discussion

In this study, we tested whether microglia contribute to impaired fear extinction after exposure to the traumatic stress of SPS, a well-studied animal model of PTSD (Lisieski et al., 2018; Ferland-Beckham et al., 2021). We found that reducing microglia with oral administration of PLX3397 prevented impaired fear extinction from developing after exposure to SPS in male rats. Although PLX3397 caused a similar reduction in hippocampal microglia in female and male rats, PLX3397 did not prevent SPS from impairing fear extinction in female rats. Consistent with previous reports suggesting that stress affects males and females differently (Keller et al., 2015; Wohleb et al., 2018), this sex-dependent effect of microglial depletion suggests that traumatic stress activates different mechanisms to cause behavioral impairment in male and female rats.

Previous studies suggested that pharmacological activation of microglia could impair fear extinction in male rats. Exposure to lipopolysaccharide (LPS), which activates microglia through their toll-like receptor 4 (Hanke and Kielian, 2011; Pardon, 2015) impaired fear extinction in male rats (Quiñones et al., 2016). Furthermore, blocking angiotensin 1 receptors that are associated with pro-inflammatory microglial activity (Labandeira-Garcia et al., 2017; Jackson et al., 2018), prevented the LPS-induced extinction deficit. Another study found that minocycline, which inhibits LPS activation of microglia (Kobayashi et al., 2013), prevented the infusion of IFNα into the rat amygdala from activating microglia and impairing fear extinction (Bi et al., 2016). Although these studies suggested that the activation of microglia impaired fear extinction, the pharmacological tools used affect cells other than microglia and female rodents were not examined.

Several studies have reported sex-dependent mechanisms associated with microglia-mediated pro-inflammatory responses which could alter their responses to PLX3397. For example, unstressed adult female rats have more microglia (Schwarz et al., 2012) and more primed microglia than male rats (Bollinger et al., 2017), suggesting that females could have more microglia after SPS which would require more PLX3397 to cause the same degree of depletion as in males. We did not find more Iba-1 positive microglia in males than females suggesting that both sexes had similar numbers of microglia prior to PLX3397 treatment. However, SPS increased microglia in the males but not in the females suggesting that exposure to the traumatic stress caused more changes in the microglia in the males.

Alternatively, since acute stress increases primed microglia in male rats and reduces them in females (Bollinger et al., 2017) and chronic stress increases microglial CSF1R expression more in male than in female mice (Wohleb et al., 2018), the acute traumatic stress of SPS could induce more activation of microglia in male rats and make them more sensitive to PLX3397. Some previous studies suggest that male microglia are more sensitive to PLX3397-mediated depletion (Berve et al., 2020; Smith et al., 2020), while others found similar sensitivity to PLX3397 in male and female rodents (Rice et al., 2015; Ma et al., 2020; Delizannis et al., 2021). In the current study, PLX3397 reduced microglia to a similar degree in both sexes suggesting that both sexes exhibited similar sensitivity to PLX3397. However, more Iba-1 positive cells were observed in the ventral hippocampus of the male rats exposed to SPS suggesting that SPS had a larger effect on microglia in the male rats. The larger effect of SPS on microglia in the males could explain why PLX3397 only prevented the impaired fear extinction in the male rats.

We also found evidence that oral PLX3397 treatment exerted sex-dependent effects on hippocampal cytokine expression. Although PLX3397 treatment did not prevent SPS-impaired extinction recall in female rats, increased hippocampal IL-1β and IL-10 cytokine expression were found in PLX3397-treated females, but not in PLX3397-treated male rats. Contrary to our expectations, we did not find alterations in pro-inflammatory cytokines in the hippocampus of SPS-exposed males or female rats. This could be due to the limitations of this study including the time of sacrifice and the lack of cell-type specificity since we examined whole hippocampal cytokine expression 1 day after behavioral procedures. In a related study, we found that isolated hippocampal microglia expressed more IL-1β and TNFα 3 days after SPS exposure (Torres-Rodriguez et al., 2022) suggesting that SPS enhances hippocampal inflammatory cytokines during the first week after exposure.

Previous work found that fear extinction varies with the estrous cycle in female rodents (Milad et al., 2009). Female rodents in the high progesterone/estrogen (proestrus) phase during extinction show better extinction recall than rats in the low progesterone/estrogen (metestrus) phase (Milad et al., 2009). Therefore, the lack of effect of PLX3397 in the females could be due to a higher proportion of animals in the metestrus phase. We did not find any difference in the extinction recall when the females were grouped based on the estrous cycle phase at the time of sacrifice, suggesting that differences in estrous phase among the groups did not obscure an effect of PLX3397 in the female rats. However, it should be noted that the estrous cycle phases were determined 2 days after extinction learning and sample sizes are insufficient to subdivide by estrous cycle phase.

It is important to note several limitations of this study. Although previous studies suggest that PLX3397 causes a global reduction in microglia (Elmore et al., 2015; Green et al., 2020), it is possible that PLX3397 failed to reduce microglia in other key structures involved in fear extinction learning and memory such as the amygdala or infralimbic cortex in the female rats. Furthermore, due to the global effect of oral PLX3397, we could not determine in which brain structure microglia were impairing extinction recall in the males. The effects observed in the ventral hippocampus provide a representation of the effects of SPS and PLX3397 on brain microglia that likely occur in many structures involved in fear regulation, since SPS also increases Iba-1 positive cells in the infralimbic cortex (Torres-Rodriguez et al., 2022) and basolateral amygdala (Lai et al., 2018). In addition, since Iba-1 expression does not distinguish between activated and quiescent microglia, the microglia remaining after depletion with PLX3397 may be more activated or functionally distinct in the female rats. Further experiments are needed to more completely understand how microglia contribute to SPS-induced behavior in male and female rodents.

In conclusion, the lack of effect of PLX3397 on the impaired fear extinction in the female rats exposed to SPS suggests that microglia play a more central role in stress-induced extinction impairment in male rodents due to sex-dependent differences in their transcriptome, proteome profile, and functionality (Guneykaya et al., 2018). Further studies are required to identify sex-dependent microglial mechanisms that explain why PLX3397 treatment only prevented SPS-induced enhanced fear acquisition and impaired fear extinction in male rats.

## Data Availability Statement

The raw data supporting the conclusions of this article will be made available by the authors, without undue reservation.

## Ethics Statement

The animal study was reviewed and approved by Institutional Animal Care and Use Committee of the Ponce Health Sciences University.

## Author Contributions

OT-R and JP designed research and wrote the article. OT-R, EO-N, YR-E, BV, and MC performed research. OT-R, EO-N, YR-E, BV, MC, and JP analyzed data. All authors contributed to the article and approved the submitted version.
