# Supplementary material for: Sex-dependent effects of microglial reduction on impaired fear extinction induced by single prolonged stress
Source: Front Behav Neurosci. 2023 Jan 9;16:1014767. doi: 10.3389/fnbeh.2022.1014767 (PMC9868263; doi:10.3389/fnbeh.2022.1014767)
Supplement: Supplementary file 1 [file Data_Sheet_1.PDF]

Figure	Sample	Statistic	Result
1B	Males, NO SPS vs SPS-only vs SPS-PLX3397 (% of freezing during AFC)	2-way ANOVA, Treatment Factor	F (2, 87) = 8.120, p = 0.0006
1B	Males, NO SPS vs SPS-only (% of freezing during COND 3-4, AFC)	posthoc, Sidak's Multiple Comparisons	t (87) = 3.157, p = 0.0195
1B	Males, SPS-ONLY vs SPS-PLX3397 (% of freezing during COND 3-4, AFC)	posthoc, Sidak's Multiple Comparisons	t (87) = 4.072, p = 0.0009
1B	Males, NO SPS vs SPS-PLX3397 (% of freezing during COND 3-4, AFC)	posthoc, Sidak's Multiple Comparisons	t (87) = 0.001022, p = 0.9999
1B	Males, NO SPS vs SPS-only vs SPS-PLX3397 (% of freezing during EXT)	2-way ANOVA, Treatment Factor	F (2, 203) = 28.75, p < 0.0001
1B	Males, SPS-ONLY vs SPS-PLX3397 (% of freezing during EXT 1-2, EXT)	posthoc, Sidak's Multiple Comparisons	t (203) = 3.417, p = 0.0160
1B	Males, SPS-ONLY vs SPS-PLX3397 (% of freezing during EXT 3-4, EXT)	posthoc, Sidak's Multiple Comparisons	t (203) = 3.154, p = 0.0382
1B	Males, SPS-ONLY vs SPS-PLX3397 (% of freezing during EXT 13-14, EXT)	posthoc, Sidak's Multiple Comparisons	t (203) = 3.616, p = 0.0079
1C	Males, NO SPS vs SPS-only vs SPS-PLX3397 (% of freezing during EXT Recall Test)	Ordinary one-way ANOVA, Treatment Factor	F (2, 29) = 16.52, p < 0.0001
1C	Males, NO SPS vs SPS-only (% of freezing during EXT Recall Test)	posthoc, Tukey's Multiple Comparisons	t (29) = 6.288, p = 0.0003
1C	Males, SPS-only vs SPS-PLX3397 (% of freezing during EXT Recall Test)	posthoc, Tukey's Multiple Comparisons	t (29) = 7.421, p < 0.0001
1C	Males, NO SPS vs SPS-PLX3397 (% of freezing during EXT Recall Test)	posthoc, Tukey's Multiple Comparisons	t (29) = 0.3497, p = 0.9669
1D	Females, NO SPS vs SPS-only vs SPS-PLX3397 (% of freezing during AFC)	2-way ANOVA, Treatment Factor	F (2, 96) = 1.105, p = 0.3355
1D	Females, NO SPS vs SPS-only vs SPS-PLX3397 (% of freezing during EXT)	2-way ANOVA, Treatment Factor	F (2, 224) = 1.358, p = 0.2593
1E	Females, NO SPS vs SPS-only vs SPS-PLX3397 (% of freezing during EXT Recall Test)	Ordinary one-way ANOVA, Treatment Factor	F (2, 32) = 5.899, p = 0.0066
1E	Females, NO SPS vs SPS-only (% of freezing during EXT Recall Test)	posthoc, Tukey's Multiple Comparisons	t (32) = 3.953, p = 0.0230
1E	Females, NO SPS vs SPS-PLX3397 (% of freezing during EXT Recall Test)	posthoc, Tukey's Multiple Comparisons	t (32) = 4.395, p = 0.0107
1E	Females, SPS-only vs SPS-PLX3397 (% of freezing during EXT Recall Test)	posthoc, Tukey's Multiple Comparisons	t (32) = 0.5289, p = 0.9260
1F	Females, DIESTRUS vs PROESTRUS vs ESTRUS vs METESTRUS (% of freezing during EXT Recall Test)	Ordinary one-way ANOVA, Column Factor	F (2, 20) = 0.7155, p = 0.5543
2B	Males, NO SPS vs SPS-only vs SPS-PLX3397 (Time in the center during OFT)	Ordinary one-way ANOVA, Treatment Factor	F (2, 29) = 2.830, p = 0.0754
2C	Males, NO SPS vs SPS-only vs SPS-PLX3397 (Total Distance Traveled during OFT)	Ordinary one-way ANOVA, Treatment Factor	F (2, 29) = 0.6343, p = 0.5375
2E	Females, NO SPS vs SPS-only vs SPS-PLX3397 (Time in the center during OFT)	Ordinary one-way ANOVA, Treatment Factor	F (2, 32) = 2.432, p = 0.1039
2F	Females, NO SPS vs SPS-only vs SPS-PLX3397 (Total Distance Traveled during OFT)	Ordinary one-way ANOVA, Treatment Factor	F (2, 32) = 2.242, p = 0.1227
3G	Males, NO SPS vs SPS-only vs SPS-PLX3397 (Number of Iba1+ Cells)	Ordinary one-way ANOVA, Treatment Factor	F (2, 20) = 11.54, p = 0.0005
3G	Males, NO SPS vs SPS-only (Number of Iba1+ Cells)	posthoc, Tukey's Multiple Comparisons	t (20), 3.660, p = 0.0444
3G	Males, SPS-only vs SPS-PLX3397 (Number of Iba1+ Cells)	posthoc, Tukey's Multiple Comparisons	t (20), 6.794, p = 0.0003
3G	Males, NO SPS vs SPS-PLX3397 (Number of Iba1+ Cells)	posthoc, Tukey's Multiple Comparisons	t (20), 3.244, p = 0.0799
3H	Males, NO SPS vs SPS-only vs SPS-PLX3397 (Iba1+ Area Fraction)	Ordinary one-way ANOVA, Treatment Factor	F (2, 20) = 7.082, p = 0.0047
3H	Males, NO SPS vs SPS-PLX3397 (Iba1+ Area Fraction)	posthoc, Tukey's Multiple Comparisons	t (20) = 3.614, p = 0.0475
3H	Males, SPS-only vs SPS-PLX3397 (Iba1+ Area Fraction)	posthoc, Tukey's Multiple Comparisons	t (20) = 5.167, p = 0.0043
3I	Females, NO SPS vs SPS-only vs SPS-PLX3397 (Number of Iba1+ Cells)	Ordinary one-way ANOVA, Treatment Factor	F (2, 20) = 15.73, p < 0.0001
3I	Females, NO SPS vs SPS-PLX3397 (Number of Iba1+ Cells)	posthoc, Tukey's Multiple Comparisons	t (20), 6.175, p = 0.0008
3I	Females, SPS-only vs SPS-PLX3397 (Number of Iba1+ Cells)	posthoc, Tukey's Multiple Comparisons	t (20), 7.504, p < 0.0001
3I	Females, NO SPS vs SPS-only (Number of Iba1+ Cells)	posthoc, Tukey's Multiple Comparisons	t (20), 1.375, p = 0.6022
3J	Females, NO SPS vs SPS-only vs SPS-PLX3397 (Iba1+ Area Fraction)	Ordinary one-way ANOVA, Treatment Factor	F (2, 20) = 3.569, p = 0.0472
3J	Females, SPS-only vs SPS-PLX3397 (Iba1+ Area Fraction)	posthoc, Tukey's Multiple Comparisons	t (20), 3.759, p = 0.0385
4A	Males, NO SPS vs SPS-only vs SPS-PLX3397 (IL-1β pg/ml)	Ordinary one-way ANOVA, Treatment Factor	F (2, 15) = 0.1574, p = 0.8558
4B	Males, NO SPS vs SPS-only vs SPS-PLX3397 (IL-6 pg/ml)	Ordinary one-way ANOVA, Treatment Factor	F (2, 15) = 0.3976, p = 0.6788
4C	Males, NO SPS vs SPS-only vs SPS-PLX3397 (TNFα pg/ml)	Ordinary one-way ANOVA, Treatment Factor	F (2, 15) = 1.347, p = 0.2897
4D	Males, NO SPS vs SPS-only vs SPS-PLX3397 (IL-10 pg/ml)	Ordinary one-way ANOVA, Treatment Factor	F (2, 15) = 0.2985, p = 0.7462
4E	Males, NO SPS vs SPS-only vs SPS-PLX3397 (IFNγ pg/ml)	Ordinary one-way ANOVA, Treatment Factor	F (2, 15) = 1.817, p = 0.1965
4F	Females, NO SPS vs SPS-only vs SPS-PLX3397 (IL-1β pg/ml)	Ordinary one-way ANOVA, Treatment Factor	F (2, 15) = 8.580, p = 0.0033
4F	Females, NO SPS vs SPS-PLX3397 (IL-1β pg/ml)	posthoc, Tukey's Multiple Comparisons	t (15), 4.996, p = 0.0080
4F	Females, SPS-only vs SPS-PLX3397 (IL-1β pg/ml)	posthoc, Tukey's Multiple Comparisons	t (15), 5.148, p = 0.0064
4G	Females, NO SPS vs SPS-only vs SPS-PLX3397 (IL-6 pg/ml)	Ordinary one-way ANOVA, Treatment Factor	F (2, 15) = 0.05001, p = 0.9514
4H	Females, NO SPS vs SPS-only vs SPS-PLX3397 (TNFα pg/ml)	Ordinary one-way ANOVA, Treatment Factor	F (2, 15) = 1.943, p = 0.1777
4J	Females, NO SPS vs SPS-only vs SPS-PLX3397 (IL-10 pg/ml)	Ordinary one-way ANOVA, Treatment Factor	F (2, 15) = 15.25, p = 0.0002
4J	Females, NO SPS vs SPS-PLX3397 (IL-10 pg/ml)	posthoc, Tukey's Multiple Comparisons	t (15), 6.993, p = 0.0005
4J	Females, SPS-only vs SPS-PLX3397 (IL-10 pg/ml)	posthoc, Tukey's Multiple Comparisons	t (15), 6.507, p = 0.0009
4K	Females, NO SPS vs SPS-only vs SPS-PLX3397 (IFNγ pg/ml)	Ordinary one-way ANOVA, Treatment Factor	F (2, 15) = 0.7512, p = 0.4888
5B	Females, AIN-76A vs PLX3397 (% of freezing during AFC)	2-way ANOVA, Treatment Factor	F (2, 66) = 0.9990, p = 0.3212
5B	Females, AIN-76A vs PLX3397 (% of freezing during EXT)	2-way ANOVA, Treatment Factor	F (2, 154) = 0.07247, p = 0.7881
5C	Females, AIN-76A vs PLX3397 (% of freezing during EXT Recall Test)	two-tailed unpaired t-test	t (22), 0.07481, p = 0.9410
5E	Females, AIN-76A vs PLX3397 (Time in the Center during OFT)	two-tailed unpaired t-test	t (22), 2.090, p = 0.0484
5F	Females, AIN-76A vs PLX3397 (Entries to the Center during OFT)	two-tailed unpaired t-test	t (22), 2.235, p = 0.0359
5G	Females, AIN-76A vs PLX3397 (Total Distance Travaled during OFT)	two-tailed unpaired t-test	t (22), 1.140, p = 0.2667

Statistical Table. Sex-dependent Effects of Microglial Reduction on Impaired Fear Extinction Induced by Single Prolonged Stress
